# Gate dependence of upper critical field in superconducting (110) LaAlO_3_/SrTiO_3_ interface

**DOI:** 10.1038/srep28379

**Published:** 2016-07-05

**Authors:** S. C. Shen, B. B. Chen, H. X. Xue, G. Cao, C. J. Li, X. X. Wang, Y. P. Hong, G. P. Guo, R. F. Dou, C. M. Xiong, L. He, J. C. Nie

**Affiliations:** 1 Department of Physics, Beijing Normal University, Beijing 100875, China; 2 Key Laboratory of Quantum Information, CAS, University of Science and Technology of China, Hefei, Anhui 230026, China

## Abstract

The fundamental parameters of the superconducting state such as coherence length and pairing strength are essential for understanding the nature of superconductivity. These parameters can be estimated by measuring critical parameters such as upper critical field, *H*_c2_. In this work, *H*_c2_ of a superconducting (110) LaAlO_3_/SrTiO_3_ interface is determined through magnetoresistive measurements as a function of the gate voltage, *V*_G_. When *V*_G_ increases, the critical temperature has a dome-like shape, while *H*_c2_ monotonically decreases. This relationship of independence between the variation of *T*_c_ and of *H*_c2_ suggests that the Cooper pairing potential is stronger in the underdoped region and the coherence length increases with the increase of *V*_G_. The result is as for high temperature superconducting cuprates and it is different than for conventional low temperature superconductors.

Oxide heterostructures have attracted much attention in recent years due to a plethora of novel or enhanced physical phenomena observed at interfaces^1^,^2^. For example, the *two dimensional electron gas* (2DEG) formed at the interface between two perovskite insulators, LaAlO_3_ (LAO) and SrTiO_3_ (STO), becomes superconducting^3^ at around 200 mK. This discovery triggered intense investigations of the superconductivity mechanism^4^,^5^,^6^,^7^,^8^ at interfaces. A number of intriguing properties emerge at the superconducting interface. Literature indicate on a pseudogap-like behavior^9^, on the electron pre-formed pairing^10^, and on the coexistence of superconductivity and ferromagnetism^11^,^12^,^13^. The phase diagram for an interface is similar to that of high temperature superconducting (HTS) cuprates^7^,^9^. The interface exhibits superconductivity for an extremely low carrier density^14^,^15^. Presented aspects may indicate on unconventional pairing mechanism. Nevertheless, the conventional electron-phonon coupling has been also considered^5^,^16^. Therefore, the microscopic mechanism responsible for interface superconductivity is still unclear and more research is of high interest.

Recently, it was reported that an energy gap persists above the superconducting dome defined as the variation of the critical temperature, *T*_c_, vs. carrier doping. Furthermore, the energy gap increases monotonically as the gate voltage *V*_G_ decreases. This correlation indicates on a pseudogap-like behavior of (001) LAO/STO interfaces^9^ and it is opposite to the *V*_G_ dependence of the superfluid density^14^. For additional information about electron pairing in interfaces, it is useful to determine fundamental superconducting parameters such as the coherence length, ξ_0_, and the strength of pairing potential. These parameters can be extracted from upper critical field, *H*_c2_; a higher *H*_c2_ means a smaller coherence length and a stronger pairing potential^17^. Previous investigations of *H*_c2_ have shown that superconductivity observed for (001) or (110) LAO/STO interfaces is of two dimensional nature^3^,^18^,^19^,^20^, but the *V*_G_ dependence of *H*_c2_ that is associated to pairing strength while being distinct from the superfluid density, has not been reported. Noteworthy is also that most studies are focused on (001) LAO/STO, while the (110) system is less explored although its study can bring an important contribution to understanding of superconductivity at interfaces^20^,^21^,^22^.

In this work we report the dependence of *H*_c2_ on *V*_G_ in a superconducting 2DEG (110) LAO/STO interface. We found that *H*_c2_ decreases when *V*_G_ increases. At the same time, the *T*_c_(*V*_G_) curve shows a dome-like shape (with a maximum). Our results indicate that the Cooper pairing potential becomes stronger in the underdoped regime.

## Results and Discussions

### Critical temperature, critical current and normal state resistance as a function of gate voltage

**Figure 1:**
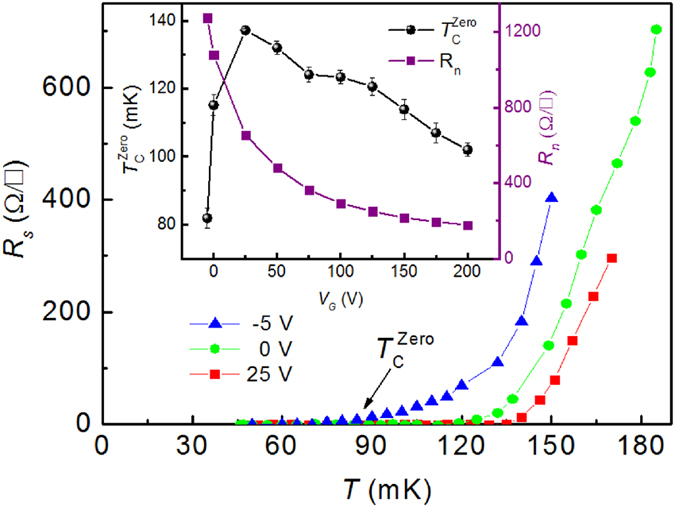
The resistance *R*_s_ of the (110) LAO/STO interface as a function of temperature at three representative gate voltages and for *B* = 0 T. The inset shows the gate voltage *V*_G_ dependence of the critical temperature 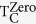 and of the normal state resistance *R*_n_. The maximum of the 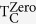 (*V*_G_) curve is at 138 mK and at 25 V. Lines are guide to the eyes.

In Fig. 1 are presented as examples, curves of (interface or sheet) resistance, *R*_s_, versus temperature for three different gate voltages (−5 V, 0 V and 25 V) under zero magnetic field. Temperature for which *R*_s_ drops below the detection limit of the measuring equipment defines the critical temperature, 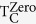 (Fig. 1). The curve of 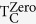 as a function of *V*_*G*_ (in zero magnetic field) has a dome-like shape with a maximum at 25 V (Fig. 1 inset). The dome-like shape of the 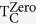 (*V*_G_) curves was previously observed in LAO/STO and (001) LaTiO_3_/SrTiO_3_ (LTO/STO) interfaces^7^,^20^,^23^. A dome-like shape with a maximum at *V*_G_ = 25 V is also obtained for the variation of the critical current *I*_c_(*V*_G_). The critical current, *I*_c_, was determined at 50 mK from *I*-*V* curves measurements (see Supplementary Information, Fig. S1). The 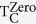
(*V*_G_) and *I*_c_(*V*_G_) similar dome-like dependencies indicate a strong influence of the carrier density on 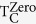 and *I*_c_.

**Figure 2:**
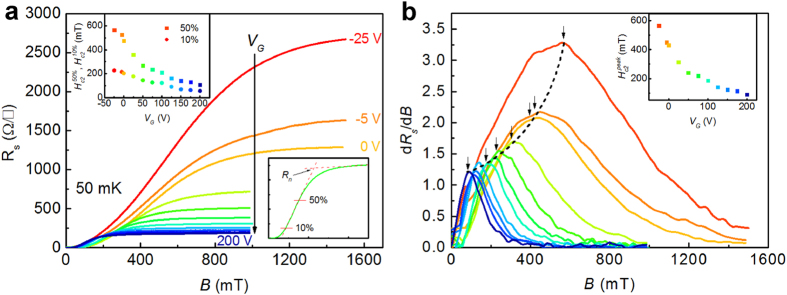
(**a**) Curves of *R*_s_(*B*) for −5 ≤ *V*_G_ ≤ 200 V at 50 mK. The inset in the right corner shows determination of *R*_n_, 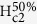 and 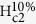. The inset in the left corner shows 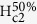
(*V*_G_) and 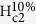
(*V*_G_) curves at 50 mK; (**b**) Curves of d*R*_*s*_/d*B* vs. *B* at 50 mK. Arrows indicate points of maximum of these curves and dashed line is guide for eyes. Magnetic field of a maximum point is defined as 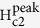. The inset shows variation of 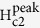 as a function of *V*_G_.

From the measurements of *R*_s_ with magnetic field (up to 1.6 T) applied perpendicular to the surface of the interface at a fixed temperature and gate voltage one can determine the normal state resistance, *R*_n_. An example of *R*_s_(*B*) curves at 50 mK and for *V*_G_ from −25 to +200 V is presented in Fig. 2a. Inset to Fig. 2a shows for selected (*T*, *V*_G_) values how *R*_n_ is determined. Namely, *R*_n_ is the resistance of the cross point between the fitting lines of the steepest part of the *R*_s_(*B*) experimental curve and of the region where *R*_s_ almost saturates. For a fixed temperature (50 mK), one *R*_n_(*V*_G_) curve is plotted in Fig. 1 inset. Enhancement of *V*_G_ decreases *R*_n_. Curve is non-linear and the decrease rate is smaller for higher *V*_G_.

It is remarkable that *V*_G_ can tune superconducting and normal state characteristics such as 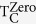 and *R*_n_, respectively. Results suggest that *V*_G_ influences 2DEG superconductivity features and the carrier density. Our results are consistent with literature^20^.

### Upper critical field as a function of gate voltage

For a fixed temperature and *V*_G_, the upper critical field *H*_c2_ is determined as the field where resistance is 10%, 50% of *R*_n_ in the *R*_s_(*B*) curves (Fig. 2a inset, right corner). This methodology was used to determine *H*_c2_ for different superconducting systems^24^,^25^,^26^. We note that magnetic field *B* is applied in this work only perpendicular to the interface surface. Reported articles^19^,^20^ indicate that *H*_c2_ shows large anisotropy when *B* is applied in-plane and out-of-plane. At 50 mK, the 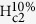
(*V*_G_) or 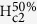
(*V*_G_) (Fig. 2 inset, left corner) curves are unexpectedly without a dome-like shape that is specific for the 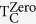
(*V*_G_) and *I*_c_(*V*_G_) (Figs 1 and S1) curves. Namely, for a lower *V*_G_, *H*_c2_ monotonically increases (Fig. 2 inset, left corner). At the same time, a decrease of *V*_G_ below *V*_G, max_ = 25 V counter-intuitively produces the decrease of both 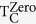 and *I*_c_ (Figs 1 and S1).

The differential curves d*R*_*s*_/d*B* as a function of *B*, obtained from *R*_*s*_(*B*) data (Fig. 2a) for different *V*_G_ and at 50 mK, are presented in Fig. 2b. Each curve displays a peak (marked with an arrow in Fig. 2b). The peak shifts to higher *B* and its intensity increases when *V*_G_ decreases. The magnetic field of the peak, denoted 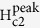, as a function of *V*_G_ (at 50 mK) is shown in Fig. 2b, inset. A dome-like shape is not obtained and, as expected, this curve has a similar behavior as 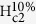
(*V*_G_) or 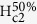. (*V*_G_) curves. Furthermore, the values of 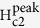 and of 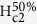 are close to each other, but we shall keep both parameters in the discussion because of their different background: *H*_c2_^50%^ is arbitrary taken, while *H*_c2_^peak^ defines the inflexion point of the *R*_s_(*B*) cur. The inflexion point may have a physical meaning and, hence, *H*_c2_^peak^ can be more sensitive to external factors than 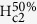.

**Figure 3:**
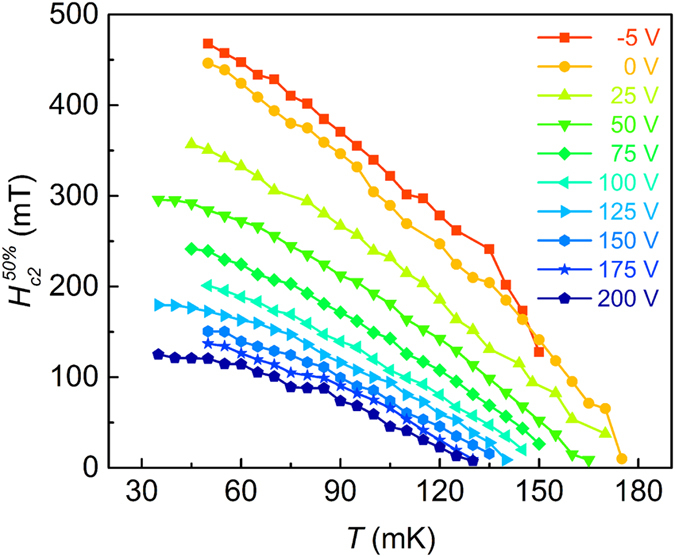
Temperature dependence of 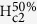 for different *V*_G_. Lines are guide for the eyes.

Figure 3 shows curves of 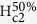
(*T*) for different *V*_G_ from −5 V up to 200 V. For all gate voltages, 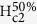 increases with temperature decrease. Curves of 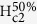. (*T*) shift into the region of higher values of 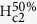-*T* for decreasing *V*_G_. This enhancement occurs even for negative *V*_G_, where a lower *V*_G_ induces a lower 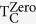. Moreover, the slope (d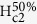/d*T*^|^_Tc_) of the 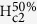
(*T*) curve approaching 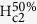
(*T*_c_) = 0 systematically increases when *V*_G_ decreases. The overall observed tendency does not change, if we use in the analysis 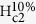 or 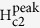 instead of 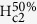 (Fig. S2 a,b). According to Ginzburg-Landau (GL) theory, near *T*_c_ the upper critical field is a linear function of (*T*_c_ − *T*) and smoothly saturates when lowering the temperature. Our results suggest an anomalous unconventional behavior of the upper critical field vs. temperature, and it is no longer appropriate to describe this dependence using the GL theory. The 
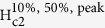
(*T* = 0 K) values obtained by linear extrapolation of e data at low temperature are increasing with decreasing *V*_G_. The determination of *H*_c2_ at *T* = 0 K lacks precision. However, if we estimate the relative *H*_c2_-increase (δ*H*_c2_) at a finite temperature (e.g. 60 mK) between the curves for *V*_G_ = −5 V and *V*_G_ = 0 V, a criterion closer to *R*_s_ = 0 shows a lower value (i.e. δ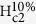 = 1.3%, while δ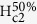 = 5.5%). This finds its understanding in the following: The emergence of *R*_s_ = 0 is due to the global superconductivity. With increasing magnetic field, a finite resistance occurs, and, hence, global superconductivity disappears. However, local superconductivity still exists before the system recovers to normal state. Thus, it is reasonable that we observe a larger *H*_*c*2_ for a more negative *V*_*G*_. This indicates that Cooper pair can persist up to a higher magnetic field than the upper critical field corresponding to global superconductivity.

### Superconductor-insulator transition

**Figure 4:**
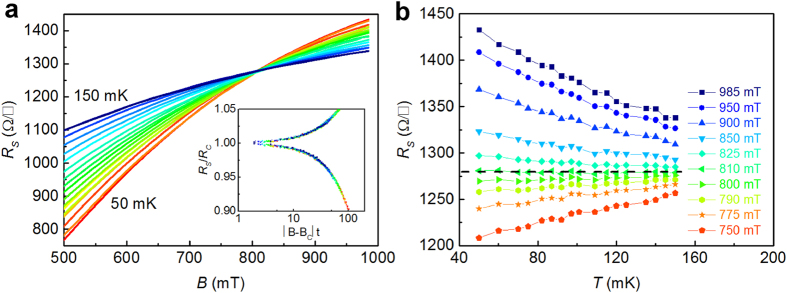
(**a**) SIM pattern: curves of *R*_s_(*B*) at different temperatures for *V*= −5 V. Note the crossing point at 810 mT that defines the *q*uantum *c*ritical *p*oint (QCP) with (*B*_c_ = 810 mT, *R*_c_ = 1297.54 Ω). The inset shows the bi-value curve obtained by collapse of the date by finite-size scaling analysis. (**b**) Curves of *R*_s_(*T*) at different magnetic fields *B* and for *V*_G_ = −5 V. Dashed line shows a plateau corresponding to QCP. Continuous lines are guide for the eyes.

The magnetic-field-induced superconductor-insulator transition(SIT)^27^ shows a characteristic fan-shaped pattern of *R*_*s*_(*B*) isotherms crossing at one point. For example, in Fig. 4a the SIT cross point for *V*_*G*_ = −5 V is at 810 mT. The *R*_*s*_(*T*) curves extracted from *R*_*s*_(*B*) show a plateau for 810 mT (Fig. 4b). The plateau separates two regimes. Therefore, the magnetic field drives a continuous quantum phase transition from a superconducting 2DEG to a weakly insulating state. Further finite-size scaling analysis shows that the data can be collapsed onto a bi-value curve (Fig. 4a, inset). It results that the crossing point is a *q*uantum *c*ritical *p*oint (QCP), at which the phase transition occurs. Literature often show magnetic-field-induced SIT for different 2D superconductors^27^,^28^, including for interfaces such as LAO/STO and (001) LTO/STO^29^,^30^.

**Figure 5:**
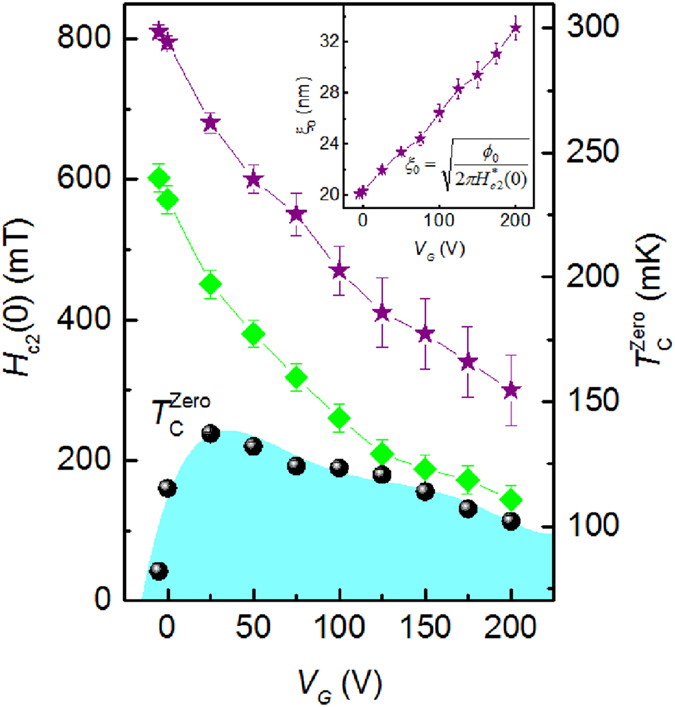
The *V*_*G*_ dependence of 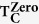, 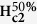 (T = 0 K) (green diamonds) and 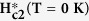. (violet stars). The inset shows the *V*_*G*_ dependence of the coherence length 
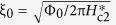
 where 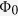 is the quantum flux. Continuous lines are guide for the eyes.

As already noted, *H*_c2_(*T*) for a fixed *V*_G_ depends on the criterion adopted for the *H*_c2_ determination in the case of a broad transition^26^,^31^ and the *H*_c2_(*T* = 0 K) cannot be determined in a reliable manner. This situation questions the intrinsic nature of the *H*_c2_ – enhancement (for a lower *V*_*G*_) in the underdoped region. Due to this, here we use another method to directly determine the zero - temperature upper critical field, 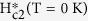. This method is independent of the criterion applied to *R*_s_(*B*) curves. At the QCP, taken as a transition point between superconducting and normal states of the 2DEG, the corresponding magnetic field is defined as 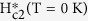. The 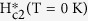 curve as a function of *V*_*G*_ is plotted in Fig. 5. Although it is considered that this method does not give accurate values of 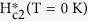
30, this barely affects our main results, the dependence on *V*_*G*_ of 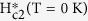 being similar to that of 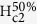
(*T* = 0 K) (Fig. 5). One reason for a non-precise determination of 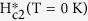 vs. *V*_G_ is: when *V*_G_ ≠ −5 V, e.g., a *V*_G_ = 75 V is used for the construction of the magnetic-field-induced SIT pattern, the crossing point of the *R*_*s*_(*B*) isotherms transforms into a field domain centered at 0.55 T and extending over ±0.05 T (Fig. S3a). In this case, the conventional power-law scaling behavior fails to describe the quantum criticality (Fig. S3b). Multiple quantum criticality was also found and reported for the (001) LTO/STO interface^29^. The phenomenon of multiple critical exponents suggests an unconventional critical behavior of SIT in the (110) LAO/STO interface, and it will be discussed elsewhere.

### Phase diagram

The superconducting phase diagram of the (110) LAO/STO interface is presented in Fig. 5. As already addressed in the previous sections, the upper critical field *H*_c2_ independently of the criterion for its determination monotonically increases when *V*_*G*_ decreases, while 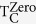 displays a dome-like curve with a maximum. In the inset to Fig. 5 is shown the GL coherence length ξ_0_ determined from *H*_c2_ as a function of *V*_*G*_. The most striking anomalous result is enhancement of *H*_c2_ accompanied by the decrease of ξ_0_ for decreasing *V*_*G*_, i.e. for the carrier depletion reflected by the dome-like curve in the underdoped region. Results indicate that the Cooper pairing potential is stronger in the underdoped region.

A systematic increase in *H*_c2_ with decreasing doping has been reported both in high-*T*_c_ cuprates and iron-based superconductors^31^,^32^. This is usually considered as an evidence for the existence of a so-called ‘pseudogap’ state, in which the bosonic pairs form above *T*_c_ but cannot condensate into superconducting state due to dilution of pairs^33^. Recently, the planar tunneling spectroscopy study in 2DEG at a (001) LAO/STO interface has shown that the energy gap ∆ increases with charge carrier depletion in both underdoped and overdoped regions^9^. And, the coherence-peak-broadening parameter *Γ* derived from the Dynes fit, that is related to the strength of the superconducting pairing interaction, increases steeply with decreasing *V*_*G*_. GuangLei Cheng *et al*.^10^ recently reported that the Cooper pairs form at temperatures well above the superconducting transition temperature of the (001)LAO/STO superconducting system. In the experiments they used a superconducting single-electron transistor and they observed that pairs condensate at low magnetic fields and temperatures. The physical understanding of the processes at (110) LAO/STO interface is analogous to that of hole-doped cuprates^31^, namely, the pairing potential is stronger and the Ginzburg-Landau coherence length *ξ*_*0*_decreases in the underdoped region as *V*_*G*_ decreases. The trend differs from that of superfluid density and superconducting transition temperature^7^,^14^. Namely, the superfluid density decreases with decreasing *V*_*G*_ providing that the phase fluctuation is important^14^,^34^,^35^. The observation of ∆/*Γ* scaled with *T*_c_ in ref. 9 implies that the limited quasiparticle lifetime controls *T*_c_ effectively^9^, and the reduction in *T*_*c*_ versus ∆ was attributed to a competing order parameter or to a weak phase coherence. In addition, in LAO/STO system the spin-orbit coupling is non negligible and strongly depends on *V*_*G*_^36^,^37^. Both conventional and unconventional pairing mechanisms have been considered to describe superconductivity in interfaces^5^,^6^,^16^,^38^. For example, the spin-orbit coupling^36^,^37^ and the coexistence of superconductivity and ferromagnetism^11^,^12^,^13^ may indicate formation of possible exotic superconducting states such as finite momentum Cooper pairing^39^,^40^. One has also to consider a different orbital reconstruction between (001) and (110) systems^20^,^41^. Superconducting properties and Rashba spin-orbit coupling can be largely tuned by controlling selective orbital occupancy in different crystal orientations^20^. For the underdoped region, it has been reported that a Lishiftz transition is observed at (001) interface^42^,^43^. The (110) system is expected to be characterized only by a 3*d*_xz_/*d*_yz_ filled electronic state; thus the superconducting properties of the (110) interface system can be substantially different from that of the (001) system.

## Conclusions

We systematically investigated the upper critical field as a function of gate voltage by ultralow temperature magnetoresistance measurements in superconducting 2DEG of a (110) LAO/STO interface. We found that upper critical field increases as the gate voltage decreases. Two independent methods to determine the upper critical field give a similar trend. This implies that the pairing potential is stronger in the underdoped region. This observation is similar to recent reports that consider a pseudogap-like behavior at the (001) LAO/STO interface. Our results for an interface with a different orientation contribute to understanding of the pairing mechanism of superconductivity at LAO/STO interface.

## Method

A five-unit-cell LaAlO_3_ thin film was grown on the (110) SrTiO_3_ substrate (500 μm thickness) by pulsed laser deposition. Details were described in ref. 19. A metallic back gate was evaporated and attached to the rear of the substrate. Leakage current was low (below the maximum value of 5 nA at V_G_ = 200 V). Standard four-terminal resistance measurements were made using wedge-bonding contacts. The sample was cooled in a dilution refrigerator with a base temperature of 10 mK. The measurement current is sufficiently low (~50 nA) to avoid sample heating at ultralow temperatures. To ensure the reversible behavior of the superconductivity, the gate voltage was ramped up to 200 V after cooling down. Perpendicular magnetic field *B* was applied to the sample (interface) surface and the field direction is the same for all measurements.

## Additional Information

**How to cite this article**: Shen, S. C. *et al*. Gate dependence of upper critical field in superconducting (110) LaAlO_3_/SrTiO_3_ interface. *Sci. Rep.* **6**, 28379; doi: 10.1038/srep28379 (2016).

## Supplementary Material

Supplementary Information
