# Supplementary material for: Gate dependence of upper critical field in superconducting (110) LaAlO3/SrTiO3 interface
Source: Sci Rep. 2016 Jul 5;6:28379. doi: 10.1038/srep28379 (PMC4932507; doi:10.1038/srep28379)
Supplement: Supplementary Information [file srep28379-s1.pdf]

Supplementary information

Gate dependence of upper critical field in superconducting (110) $LaAlO_3/SrTiO_3$ interface

S. C. Shen,[1] B. B. Chen,[2] H. X. Xue,[1] G. Cao,[2] C. J. Li,[1] X. X. Wang,[1] Y. P. Hong,[1] G. P. Guo,[2*] R. F. Dou,[1] C. M. Xiong,[1] L. He,[1] and J. C. Nie[1*]

[1]Department of Physics, Beijing Normal University, Beijing 100875, China.
[2]Key Laboratory of Quantum Information, CAS, University of Science and Technology of China, Hefei, Anhui 230026, China.

*Correspondence to: jcnie@bnu.edu.cn, gpguo@ustc.edu.cn.

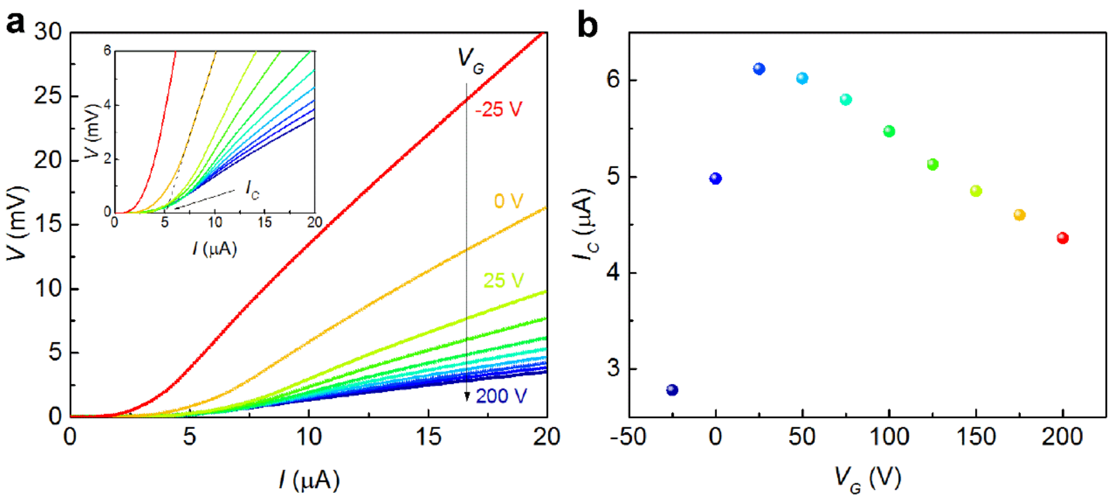

Supplementary, Figure S1 (a) - The *I-V* curves measured at 50 mK for V_G from -25 V to 200 V. The inset shows the same data and procedure to determine I_c; (b) – The dependence of I_c on V_G. Note the dome-like shape similar to that of the $T_C^{Zero}(V_G)$ curve from Fig. 1 inset.

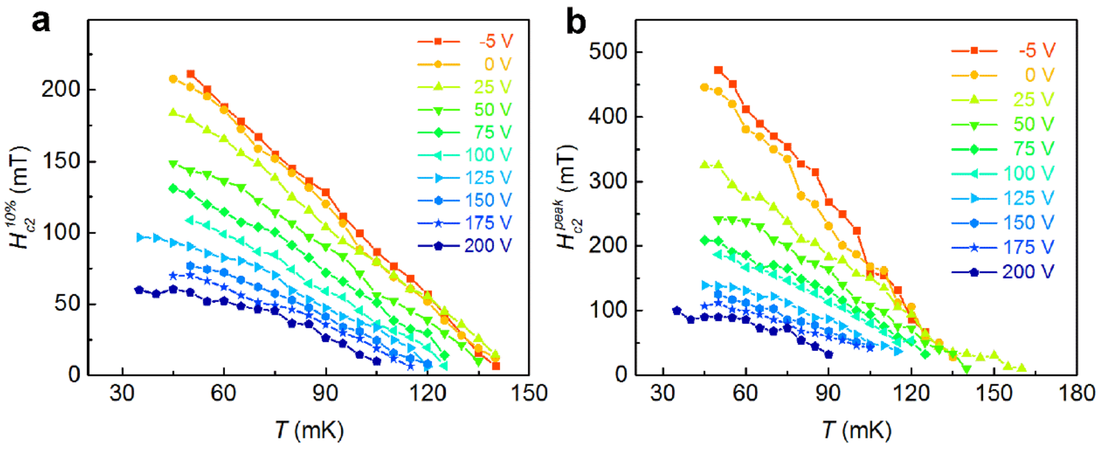

Supplementary, Figure S2 Temperature dependence of the upper critical fields: (a)- $H_{c2}^{10\%}$ and (b) - H_{c2}^{peak}.

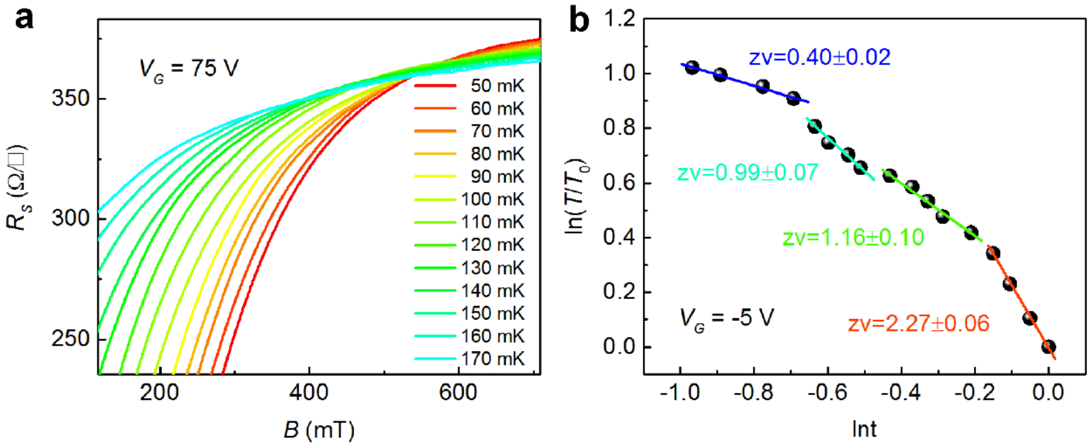

Supplementary, Figure S3 (a) – SIM pattern: curves of $R_s(B)$ at different temperatures for V_G = 75 V. Note the crossing points domain. (b) – critical parameters obtained from the power-law scaling showing multiple values (V_G = -5 V). Continuous lines are guide for the eyes.

1. Goldman, A. Superconductor-insulator transitions. *Int. J. Mod. Phys. B* **24**, 4081-4101 (2010).

2. Biscaras, J., Bergeal, N., Hurand, S., Feuillet-Palma, C., Rastogi, A., Budhani, R. C., *et al.* Multiple quantum criticality in a two-dimensional superconductor. *Nat. Mater.* **12**, 542-548 (2013).
